# Cause-specific neonatal mortality in a neonatal care unit in Northern Tanzania: a registry based cohort study

**DOI:** 10.1186/1471-2431-12-116

**Published:** 2012-08-07

**Authors:** Blandina Theophil Mmbaga, Rolv Terje Lie, Raimos Olomi, Michael Johnson Mahande, Gunnar Kvåle, Anne Kjersti Daltveit

**Affiliations:** 1 Kilimanjaro Christian Medical Centre and Kilimanjaro Christian Medical College, P.O. Box 3010, Moshi, Tanzania; 2 Department of Public Health and Primary Health Care, University of Bergen, P.O. Box 7804, Bergen 5020, Norway; 3 Centre for International Health, University of Bergen, P.O. Box 7804, Bergen, Norway; 4 Division of Epidemiology, Norwegian Institute of Public Health, Oslo, Norway

**Keywords:** Neonatal mortality, Neonatal deaths, Neonatal morbidity, Birth asphyxia, Prematurity, Causes of death

## Abstract

**Background:**

The current decline in under-five mortality shows an increase in share of neonatal deaths. In order to address neonatal mortality and possibly identify areas of prevention and intervention, we studied causes of admission and cause-specific neonatal mortality in a neonatal care unit at Kilimanjaro Christian Medical Centre (KCMC) in Tanzania.

**Methods:**

A total of 5033 inborn neonates admitted to a neonatal care unit (NCU) from 2000 to 2010 registered at the KCMC Medical Birth Registry and neonatal registry were studied. Clinical diagnosis, gestational age, birth weight, Apgar score and date at admission and discharge were registered. Cause-specific of neonatal deaths were classified by modified Wigglesworth classification. Statistical analysis was performed in SPSS 18.0.

**Results:**

Leading causes of admission were birth asphyxia (26.8%), prematurity (18.4%), risk of infection (16.9%), neonatal infection (15.4%), and birth weight above 4000 g (10.7%). Overall mortality was 10.7% (536 deaths). Leading single causes of death were birth asphyxia (n = 245, 45.7%), prematurity (n = 188, 35.1%), congenital malformations (n = 49, 9.1%), and infections (n = 46, 8.6%). Babies with birth weight below 2500 g constituted 29% of all admissions and 52.1% of all deaths. Except for congenital malformations, case fatality declined with increasing birth weight. Birth asphyxia was the most frequent cause of death in normal birth weight babies (n = 179/246, 73.1%) and prematurity in low birth weight babies (n = 178/188, 94.7%). The majority of deaths (n = 304, 56.7%) occurred within 24 hours, and 490 (91.4%) within the first week.

**Conclusions:**

Birth asphyxia in normal birth weight babies and prematurity in low birth weight babies each accounted for one third of all deaths in this population. The high number of deaths attributable to birth asphyxia in normal birth weight babies suggests further studies to identify causal mechanisms. Strategies directed towards making obstetric and newborn care timely available with proper antenatal, maternal and newborn care support with regular training on resuscitation skills would improve child survival.

## Background

The aim of the United Nations’ Millennium Development Goal 4 (MDG4) is to reduce under-five mortality worldwide to 30 deaths per 1000 live births by 2015. Globally, an estimate of 10.6 million children under five years died in 2000 [1], declining to 8.8 million in 2008 [2] and further to 7.7 million in 2010 [3]. At the same time, methods of estimation and available sources of information for under-five mortality have improved [1-4]. However, share of neonatal deaths increased from 37% in 2000 [1,5] to 41% in 2008 [2]. The slow decline in neonatal mortality as compared to post-neonatal mortality calls for attention and efforts to reverse this trend. Within the neonatal period an estimated 50% of all deaths are within the first 24 hours while 75% are within the first week of life [5]. Given that a large fraction of these deaths are preventable, a focus on mortality in the first week of life is important in order to accelerate the millennium goal.

Worldwide, the most important single causes of neonatal deaths are preterm birth, birth asphyxia, sepsis and pneumonia [2,5,6]. This reflects the mortality pattern in low income countries where neonatal mortality is high, whereas, in high income countries where mortality is low, preterm birth and congenital malformations are the leading causes of death [7]. The World health organization (WHO) estimates that birth weight below 2500 g indirectly contributes to about 15% of the neonatal mortality, ranging from 6% in high income countries to 30% in low income countries, with preterm birth and related complications being the underlying cause [8].

Tanzania is among the countries with insufficient progress towards achieving the MDG4 showing slow decline in under-five mortality rate [4]. Neonatal mortality in Tanzania is estimated to account for 28-34% of deaths in children younger than five years [2,3,9], with the most frequent single causes of death being prematurity, birth asphyxia, sepsis and pneumonia [2,10].

Globally, it has been estimated that ninety nine percent of neonatal deaths occur in developing country where also vital registration is limited [5]. The UNICEF report in 2002 estimated that only 5% of Tanzanian children aged five years are registered, with marked difference in percentage between rural (3%) and urban (22%) areas [11]. A recent country survey in Tanzania reported consistently low registration rate for under-five years children (16%), of whom about half (8%) receive birth certificate [12]. Also this survey indicated the difference in the birth registration rate between rural (10%) and urban (44%) areas.

Identification of cause-specific mortality in a particular setting is important to design interventions directed to improve neonatal survival. Therefore, in order to address neonatal mortality in Tanzania and possibly identify areas of prevention, we studied causes of neonatal admission and cause-specific neonatal mortality in a cohort of neonates delivered at the KCMC tertiary hospital and admitted to NCU.

## Methods

### Setting

The primary study unit was the neonatal care unit (NCU) at the Kilimanjaro Christian Medical Centre (KCMC) in Northern Tanzania. As a tertiary and a referral hospital for the four regions in the Northern part of the country, the NCU at KCMC receives high risk babies delivered within the institution and referrals from other health facilities or from home. The NCU has bed capacity of 40 babies. The babies are nursed in locally made baby cots and there are heaters to keep the room as well as the babies warm. The unit has no mechanical ventilator or any continuous positive airway pressure machine. The babies receive oxygen through nasal prongs or face masks. There is no central oxygen supply; instead oxygen is usually stored in big cylinders. In addition, there are four portable oxygen concentrators. The midwives in the labour ward are responsible for the initial resuscitation of normal deliveries. Doctors from the paediatric department are called to the labour ward to attend the at risk pregnancies. Normal babies are put on the breast as soon as they recover from the birth trauma which is usually within the first four hours after delivery. Babies in the nursery rooms are either fed on expressed breast milk if the mothers are around or formula in the absence of the mothers. Feeding is given either through nasal gastric tube, syringe or cup and spoon. For neonates with feeding difficulties, parenteral feeding with either 10% or 5% dextrose depending on the gestational age is used.

The neonatal care unit has 3 nursery rooms, one for preterm babies, one for term babies with non-infectious conditions and the third is reserved for bacterial as well as viral infections. Kangaroo mother care is being encouraged in the unit and a special room has been allocated for that purpose. Two other rooms are available for stable babies who are given to their mothers and to continue treatment under the supervision of the unit nurses, pending discharge when fully recovered.

We have previously reported that approximately15% of the births within the KCMC hospital were transferred to neonatal care unit for admission [13]. Criteria for transfer to neonatal care unit were found to be low Apgar score, prematurity, birth weight <1800 or birth weight >4000 g, congenital malformation, suspected infection or risk of infection, as well as for observation in case of poor maternal outcome or poor obstetric history.

### Study design and subjects

This is a birth registry based cohort study. Neonates registered in the Medical Birth Registry and admitted to the neonatal care unit at KCMC, were included. The KCMC Medical Birth Registry was established in 1999 as collaboration between Kilimanjaro Christian Medical College, Tumaini University and the University of Bergen, Norway. The Medical Birth Registry and neonatal registry were officially established in July 2000.

From July 2000 to October 2010, a total of 34087 births were registered in the birth registry, of which 5033 (14.8%) neonates were admitted to the neonatal care unit. These neonates formed our study population.

### Data collection

Information from all mothers who delivered at KCMC was collected within the first 24 hours after delivery based on a standardized questionnaire as well as antenatal records. Detailed information collected for the Medical Birth Registry are described elsewhere [13].

The neonatal registry form was recorded for all neonates who were transferred for admission in the NCU. The data collection form was administered by a trained paediatric nurse who worked in a neonatal care unit. The collection of information for neonates admitted to the NCU was done during admission and finalized after discharge or death. Information collected in the neonatal registry includes child’s ID-number, date of admission, reasons for admission, investigations done, management given, clinical details at discharge and death reports. The information collected from the Medical Birth Registry and neonatal registry were finally entered into a computerized database where they can be linked using the child’s ID-number.

On arrival in the NCU, the baby is examined by a medical officer. Estimation of the gestational age is done using the Finnstrøm maturity score, which is a simple method for assessing maturity based on external characteristics. Tables for maturity score and transformation to gestational age are included in the KCMC Paediatric management schedule [14]. The diagnosis of birth asphyxia is based on Apgar score below 7 at five minutes and severity of neonatal condition at admission, based on the presence of convulsions within the first 24 hours. A diagnosis of infection is based on clinical symptoms and signs, evidence of focal lesions, or laboratory findings including blood cultures. Thin and thick blood slides are taken in order to rule out congenital malaria if suspected. A diagnosis of congenital malformation is based on the child’s clinical presentation at admission and screening with the aid of X-ray, ultrasound scan, echocardiography and CT scan when applicable. Up to three clinical diagnoses are recorded for each neonate. The two conditions Respiratory Distress Syndrome (RDS) and Necrotizing Enterocolitis (NEC) were frequently reported as preterm complications and were mainly diagnosed clinically followed by X-ray investigation. Other preterm complications such as Intraventricular haemorrhage (IVH) may be underreported in the clinical discharge or death diagnosis reports as brain ultrasound investigation is not routinely done for preterm babies. Management of all admitted neonates follows the KCMC Paediatric Management Schedules [14]. This book was prepared by past and present paediatricians in the department by adapting the WHO guidelines for management of common illnesses in limited resource settings [15]. During the study period there was no documented change in clinical practice and management specifically for prematurity and birth asphyxia. As for neonatal infections, the department observed a high rate of staphylococcal isolates from newborns blood culture sensitive to cloxacilline and gentamycine in early 2000 also observed and recommended by Klingenberg et al., [16]. Therefore, treatment guidelines for neonatal infection changed from gentamycine and ampicilline to gentamycine and cloxacilline [14]. Surfactant treatment is not available in the neonatal unit at the moment. Therefore, babies with RDS are managed as severely preterm babies with oxygen therapy, thermal care, nutritional support, antibiotic treatment and vitamin K [14]. In addition, aminophylline is given for babies with apnoea attacks not responding to tactile stimulation.

### Data analysis

We first coded clinical diagnoses independently depending on the presence of any diagnosis; a neonate having more than one diagnosis was recorded in different categories in order to study all causes of admission, case fatality and their contribution to total neonatal mortality. Case fatality was computed as number (percentage) of deaths within each clinical discharge diagnosis among admitted babies with the same condition.

Secondly, the Wigglesworth classification with the revised decision tree [17,18] with modifications from the Neonatal and Intrauterine deaths Classification according to Etiology (NICE) [19] was used to classify neonatal deaths in a hierarchical order. The criteria described in Table 1, adapted and modified from elsewhere [20] were used in order to assign babies to a single cause of death. Fourteen neonates who had symptoms recorded as discharge/death diagnosis were reassigned a cause of death after reviewing the birth history and clinical presentation. Finally, in hierarchical order, each neonatal death was classified into one of the five single causes of death groups; 1) congenital malformation 2) prematurity 3) birth asphyxia 4) infection and 5) other causes of death. The single causes of death were then stratified into 5 birth weight categories in order to study birth weight specific mortality.

**Table 1:** Criteria used for case definition to identify cause-specific mortality*

**Causes of death**	**Case definition**	**Search criteria for clinical diagnosis in registry**
Congenital malformation	lethal congenital malformation (congenital heart, spinal bifida, congenital syndromes, gastrointestinal malformation)	− multiple congenital malformation
		− congenital heart disease
		− spinal bifida/hydrocephalus
		− congenital syndromes or syndrome baby and deaths due to systemic conditions such as-renal failure or gastrointestinal system
Birth asphyxia	birth asphyxia, hypoxic ischaemic encephalopathy Apgar based definition	− birth asphyxia weight >1000 g or gestational age >27 weeks
		− birth asphyxia and prematurity with gestational age ≥33wks and birth weight ≥2500 g or birth weight ≥ 1800 g if gestational age unknown
		− hypoxic ischaemic encephalopathy five minutes Apgar less than 7
Prematurity	prematurity, respiratory distress syndrome in preterm, necrotizing enterocolite in preterm birth	− prematurity
		− prematurity and asphyxia with gestational age < 33 weeks and birth weight < 2500 g or birth weight < 1800 g if gestational age is unknown
		− respiratory distress syndrome in preterm
		− necrotizing enterocolitis
		− birth asphyxia gestational age <27 weeks or birth weight <1000 g
		− infection with gestational age <33 weeks
Infection	neonatal infection, sepsis/septicaemia, meningitis, pneumonia	− neonatal infection
		− sepsis/septicaemia
		− meningitis
		− pneumonia
		− impetigo neonatorum
Others	other specific causes not classified above	− neonatal jaundice
		− meconium aspiration syndrome
		− respiratory distress syndrome in term babies

*The criterion was adapted from modified Wigglesworth classification and the revised decision tree [17,18], and NICE classification [19] as used by Lawn et al. in classifying causes of neonatal deaths [20] with minor modifications.

Data were analyzed using Statistical Package for Social Science (SPSS) program Version 18.0 for Window (SPSS 18.0 Chicago Inc. III, USA). Descriptive statistics measures such as mean, standard deviation, rate and proportions were calculated.

### Ethical approval

The birth registry at Kilimanjaro Christian Medical Centre obtained ethical clearance from the Tanzania Ministry of Health, Institute of Science and Technology, from the Norwegian National ethics committee and from the Kilimanjaro Christian Medical College (KCM-College) research ethics committee in 1999. Informed consent was obtained from mothers prior to the interview. The protocol for this study was approved by KCM-College research ethics committee, with certificate no. 333 of 15^th^ July 2010.

## Results

### Characteristics of mothers and neonates

A total of 5033 inborn neonates were admitted, of these 2806 (55.8%) were males, (Table 2). Most of them 4583 (91.1%) were singletons. The mean (SD) birth weight and gestational age were 2899 (857) grams and 38 (3.5) weeks, respectively. Babies with missing data on birth weight and gestational age accounted for (0.8% and 9.1%) of all admissions, respectively. The majority of mothers 4871 (96.8%) had booked for antenatal care, and 49.0% of the babies were born after caesarean section. The mean (SD) maternal age and number of antenatal care (ANC) visits were 27.6 (6.2) years and 4.3 (2.0) visits, respectively.

**Table 2:** Characteristics of admitted neonates

**Characteristics**	**n (%)**	**Characteristics**	**Mean (SD)**
**Child**		**Child**	
Males	2806 (55.8)	Birth weight	2899 (857) gm
Female	2227 (44.2)	Birth length	47.5 (3.6) cm
Singletons	4583 (91.1)	Gestational age	38.0 (3.5) wks
From labour/obstetric ward	4788 (95.1)	**Maternal**	
From home/other facilities	245 (4.9)	Mean maternal age	27.6 (6.2) years
Born by caesarean section	2468 (49.0)	Mean ANC visits	4.3 (2.0)
Low birth weight (<2500 g)	1459 (29.0)		
Gestational age <37 weeks	1178 (25.8)		
**Maternal**			
Below 18 years	136 (2.7)		
18-25 years	1879 (37.3)		
26-35 years	2450 (48.7)		
Over 35 years	558 (11.1)		
First born	2119 (43.6)		
Single mother	60 (12.5)		
Antenatal care booking	4871 (96.8)		

### Causes of admission

The leading causes of admission were birth asphyxia 1351 (26.8%), prematurity 930 (18.4%), risk of infection 852 (16.9%), neonatal infection 776 (15.4%), and large for gestational age (birth weight above 4000 g) 538 (10.7%), (Table 3). Of those admitted 1459 (29.0%) were low birth weight (<2500 g) and 1178 (25.8%) were born before 37 weeks of gestation.

**Table 3:** Clinical discharge diagnosis, case fatality and contribution to overall mortality in a neonatal care unit (N = 5033). Ranked according to case fatality

Diagnosis**	**Admitted**	**Case fatality**	**Proportion of deaths**
	**n (%)**	**n (%)**	**(%)**
RDS	99 (2.0)	52 (52.5)	9.7
Congenital malformation	111 (2.2)	49 (44.1)	9.1
Asphyxia	1351 (26.8)	321 (23.8)	59.9
Prematurity	930 (18.4)	208 (22.4)	38.8
MAS	103 (2.0)	8 (7.9)	1.5
Neonatal infection	776 (15.4)	58 (7.5)	10.8
Other conditions	230 (4.6)	8 (3.5)	1.5
Neonatal jaundice	174 (3.5)	5 (2.9)	0.9
Risk of infection	852 (16.9)	11 (1.3)	2.1
LGA (>4000 g)	538 (10.7)	5 (0.9)	0.9
Normal for observation	368 (7.3)	0	0
TTN	132 (2.6)	0	0
**Growth characteristics**			
Birth weight <2500 g	1459 (29.0)	279 (19.1)	52.1
Gestational age <37 weeks	1178 (25.8)	217 (18.4)	40.5

**A neonate having more than one diagnosis is counted more than once; therefore proportion of deaths exceeds 100%. Abbreviations: *LGA* (Large for gestational age), *MAS* (meconium aspiration syndrome), *RDS* (Respiratory Distress Syndrome), *TTN* (Transient Tachpnoea of Newborn).

### Case fatality

**Figure 1:**
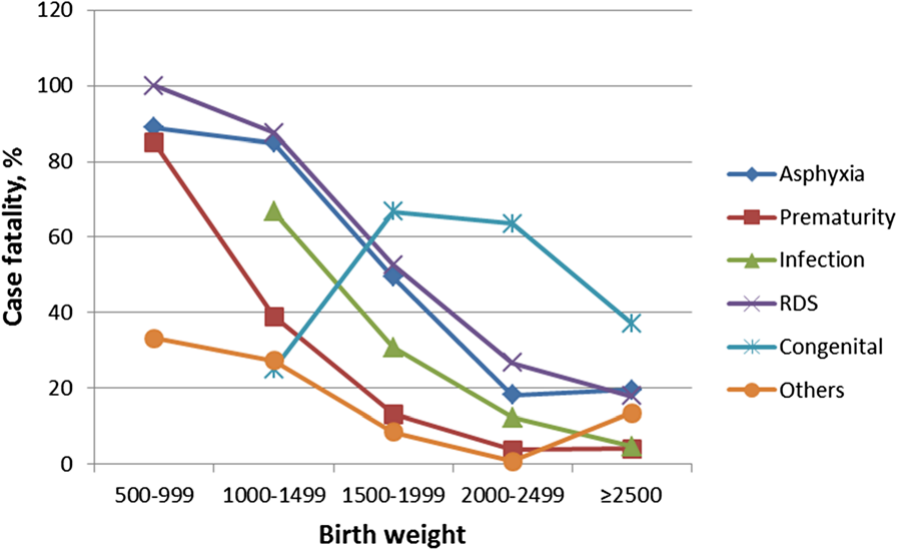
Case fatality according to clinical discharge diagnosis, in relation to birth weight (N = 5033 neonates).

Among the admitted neonates, 536 (10.7%, 95% CI 9.8 - 11.6) died. High case fatality was observed for babies with Respiratory Distress Syndrome (RDS), 52 (52.5%), congenital malformations 49 (43.6%), birth asphyxia 321 (23.8%) and prematurity 208 (22.4%), (Table 3). Large for gestational age babies had the lowest case fatality (0.9%) in which the five cases that died had birth asphyxia as the underlying cause. Except for congenital malformations, case fatality decreased with increasing birth weight (Figure 1).

### Cause-specific neonatal mortality

**Figure 2:**
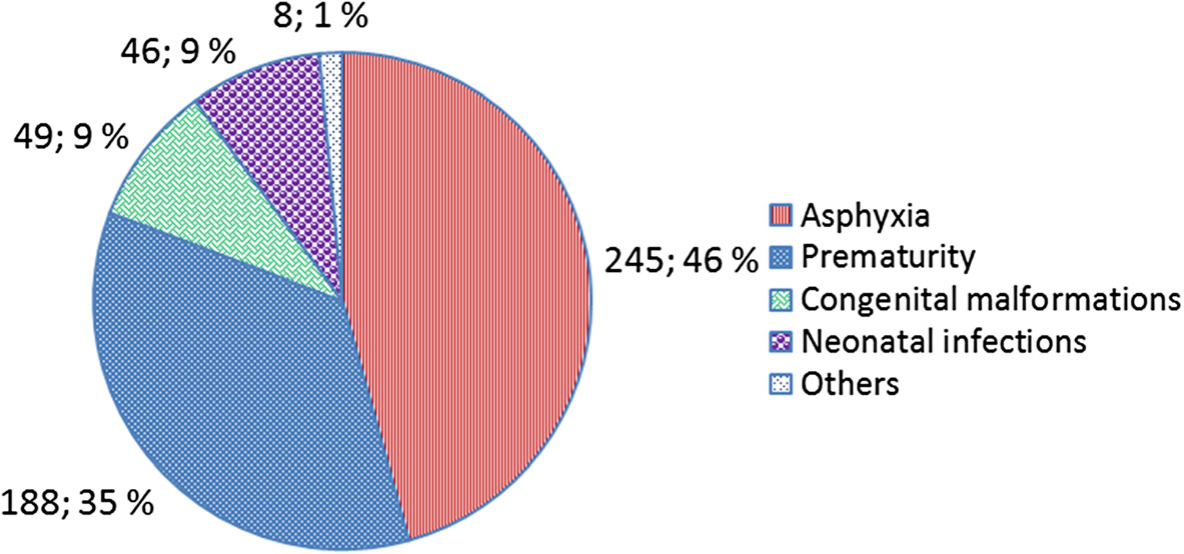
Single causes of neonatal deaths in a NCU (N = 536 neonatal deaths).

**Figure 3:**
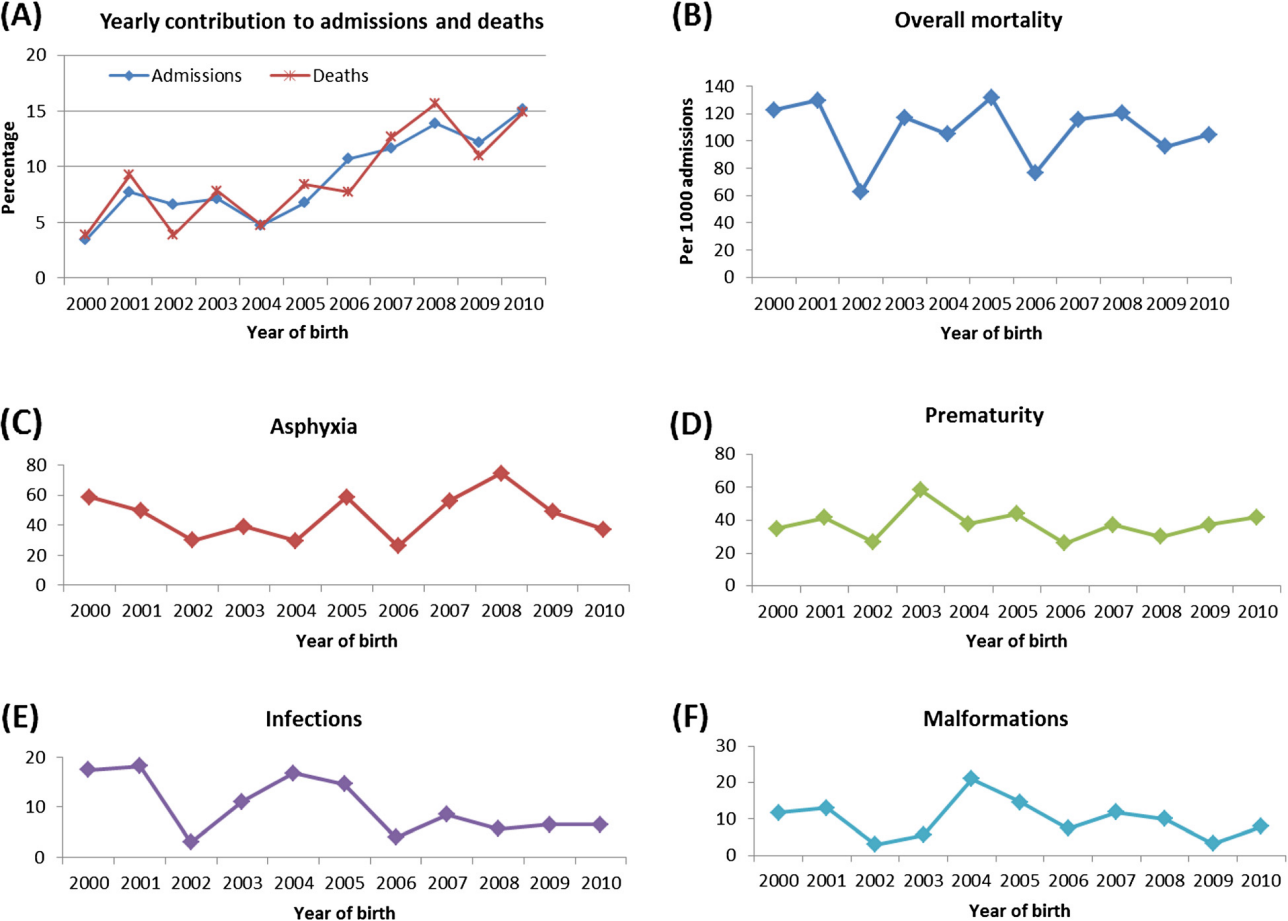
Graphs showing percentage contribution to overall admissions and overall deaths for each year (A), trend in death rate per 1000 admissions according to year of birth for overall (B) and in each category of cause-specific of deaths (C-F).

The leading single causes of neonatal death were birth asphyxia 245 (45.7%), prematurity 188 (35.1%), congenital malformations 49 (9.0%), and infections 46 (8.6%), (Figure 2). During the study period, there was no clear trend observed over time for overall mortality or within each cause of death (Figure 3).

The mean (SD) for gestational age and birth weight for premature babies who died was 30.3 (3.3) weeks and 1342 (457) grams, respectively. On the other hand, the mean (SD) for babies who died due to birth asphyxia was 38.7 (3.0) weeks and 2843 (705) grams, respectively. Of the babies who died due to asphyxia 143 (58.4%) had 5-minute Apgar score below 5 whereas, 102 (41.6%) had 5-minute Apgar score above 7. The majority of deaths attributed to asphyxia (179/245, 73.1%) occurred in normal birth weight babies (Table 4). Birth asphyxia in normal birth weight babies and prematurity in low birth weight babies each contributed to about one third of all deaths; 179/536 (33.4%) and 178/536 (33.2%), respectively. Mortality decreased with increasing birth weight.

**Table 4:** Single causes of neonatal death by birth weight (N = 536)

		**Birth weight in grams**					
**Causes**	**Total deaths**	**500- 999**	**1000- 1499**	**1500- 1999**	**2000- 2499**	**2500+**	**Unknown**
No. admitted	5033	43	254	552	612	3535	37
Congenital	49	0	1	4	14	29	1
Prematurity	188	37	85	52	4	3	7
Asphyxia	245	0	11	19	34	179	2
Infection	46	0	4	6	6	29	1
Other	8	0	0	0	2	6	0
**Total deaths**	**536**	**37**	**101**	**81**	**60**	**246**	**11**
(% ) deaths	(10.7)	(86.0)	(39.8)	(14.7)	(9.8)	(7.0)	(29.7)

Most babies (304, 56.7%) died within the first 24 hours, and as many as 491 (91.6%) within the first week (Table 5). Birth asphyxia and prematurity accounted for 86.8% of all deaths within the first 24 hours.

**Table 5:** Single causes of neonatal deaths in relation to time of death (N = 536)

**Diagnosis**	**Total**	**Day 0-1**	**Day 2-7**	**After day 7**
Congenital	49	23	20	6
Prematurity	188	109	59	20
Asphyxia	245	152	87	6
Infection	46	16	17	13
Other	8	4	4	0
**Total (%)**	**536 (100.0)**	**304 (56.7)**	**187 (34.9)**	**45 (8.4)**

## Discussion

The motive of our study was to identify morbidity and causes of neonatal death of inborn neonates admitted to a neonatal care unit at a tertiary hospital in Northern Tanzania. Our main results are largely consistent with the global pattern of mortality [2,5], with birth asphyxia, prematurity, and infection as the most important single causes of neonatal death. Our results should be interpreted bearing in mind that it includes only inborn neonates delivered at a tertiary health facility. Neonates who are admitted at a tertiary hospital NCU represent the important subgroup of neonates who have high risk of morbidity and mortality.

### Overall mortality

Overall, 10.7% of the admitted neonates died in the neonatal period. This is similar to a study done in a regional referral hospital in Sudan where also only inborn neonates were included [21]. Most other studies include both outborn and inborn neonates, where in general mortality is higher [22-24]. A previous study from KCMC NCU in 2003 including both inborn and outborn neonates reported 19% neonatal mortality [16]. A study from Nigeria reported overall mortality of 25.5%; 20.3% among inborn neonates and 64.2% among outborn neonates [22]. A study from Bangladesh [23] reported 8% mortality in inborn neonates and 25.6% in outborn neonates. The variations in mortality probably reflect local and national differences in care pattern of newborn babies.

### Causes-specific mortality

Our finding that birth asphyxia was the leading cause of death is consistent with a previous study from a university and tertiary care hospital in Tanzania [10]. In contrast, the global pattern and studies from university and tertiary care hospitals find prematurity to be the leading cause of death [5,22-24]. One explanation of the high number of deaths due to asphyxia in our data may be the definition criteria for asphyxia that we used, which included some of the preterm babies. In some studies [21,24], all preterm babies who die are classified with prematurity as cause of death. Of particular interest is the high number of deaths attributable to asphyxia in normal birth weight infants in our study (one third of all deaths) because they may represent a potential for prevention. Basic training on newborn resuscitation skills and proper newborn resuscitation immediate after birth has proved to reduce mortality among babies born with birth asphyxia up to 40% [25-27]. A recent study in six developing countries showed that training on Essential Newborn Care which includes training on basic resuscitation had no effect on early neonatal mortality. However, there was a significant reduction in the rate of stillbirths primarily fresh, most likely as an effect of resuscitation of babies who would have been misclassified as stillbirths before training [28]. On the other hand, there was no additional effect of training in the Neonatal Resuscitation Program once the Essential Newborn Care training was already in place [28]. Training on newborn resuscitation immediately after birth is highly needed in Tanzania, where only 16% of health care services reported that they offer newborn respiratory support [29].

Prematurity was the second most important cause of death. Management of premature babies requires high specialized equipment, highly trained personnel and financial support [26,30]. In high income countries where ventilation technology and the use of surfactant have been implemented, the survival of premature babies has improved [30]. RDS is a known very frequent complication of preterm babies due to lung immaturity, and babies with RDS had the highest case fatality in our study, which is also reported elsewhere [16,24,31,32]. The high case fatality in babies with RDS reflects the inadequate care of these neonates in developing countries [30,33].

Some specific and simple measures has been identified which could be implemented to reduce deaths related to low birth weight and preterm in low income countries [27,34,35]. These include among others prophylactic use of steroid during premature labour, antibiotic for premature rupture of membrane, early breast feeding, treatment of infection, hospital-based kangaroo mother care, prevention of hypothermia, feeding and nutritional support. A recent meta-analysis review found hospital-based Kangaroo mother care (skin-to-skin contact) implemented within the first week of life for stable preterm and low birth weight neonates was effective and could reduce neonatal mortality up to 51% [36].

Mortality due to infection was low compared to the global pattern as well as the pattern in low income countries [10,16,23,25]. The low number of deaths due to infection might in part be explained by the inclusion of only inborn neonates, since appropriate treatment of infection or suspected infection can start with a minimum of time delay after delivery.

In a previous study from the same NCU where both inborn and outborn neonates were included one fifth of the deaths were due to infection [16]. Furthermore, the use of Gentamycin and Cloxacillin instead of Gentamycin and Ampicillin [14] introduced in the early 2000 for neonatal infections in the department may have played a role in increased survival in infected neonates. A similar change in antibiotic treatment in Nigeria resulted in a 32% reduction in mortality associated with septicaemia [25]. The routine transfer to NCU of all neonates at risk of infection or suspected infection due to premature/prolonged rupture of membrane for antibiotic prophylaxis [13], might also have contributed to low mortality due to infection in this setting. We have previously shown that babies of mothers with premature/prolonged rupture of membrane had a 2 fold risk of being transferred to NCU for antibiotic prophylaxis due to risk of infection [13].

The majority of women in low income countries do not access early ultrasound scan for screening of congenital malformation, and there are very few early terminations of pregnancies due to severe/fatal congenital malformations. Availability of management/surgery for neonates with severe congenital malformations is limited, and under the prevailing circumstances we suggest that few of these deaths could have been prevented.

### Time of death

The majority of neonatal deaths occurred during the first day after admission and more than 90% within the first week of life. We found that birth asphyxia and prematurity were the major causes of death within the first 24 hours, whereas deaths related to infections were more frequent after first week. These results are similar to what has previously been observed [25,34].

## Strengths and limitations

This study used a large Medical Birth Registry with a complementary neonatal registry of neonates admitted to NCU where data are collected using standardized questionnaires. The data set contains a substantial number of neonates to be studied. All admitted neonates were given at least one discharge diagnosis and all information from the NCU could be linked to their information recorded in the birth registry. The modified Wigglesworth classification used to classify single causes of neonatal death was selected because it is simple to apply and since causes of death according to this classification have clear implications for clinical management. The classification gives the opportunity to identify areas of health care provision in need of prevention or improvement in management and care to improve neonatal survival. Since KCMC is a tertiary care hospital and included only inborn neonates, the results may not be generalized to all hospitals in Kilimanjaro or to the population.

## Conclusions and recommendations

Birth asphyxia, prematurity and infection were the major single causes of neonatal morbidity and mortality. Birth asphyxia in normal birth weight babies and prematurity with low birth weight, each accounted for one third of all deaths, and some of these deaths may be preventable. First, strategies directed towards strengthening screening and identification of mothers at risk and early referral mechanisms for care and support is needed. Second, regular and continuous training of health personnel on essential newborn care to ensure basic knowledge on resuscitation skills and immediate actions needed for asphyxiated newborns should be strengthened. Care of premature babies should include in hospital kangaroo mother care for stable preterm and low birth weight babies, thermal care, feeding and nutritional support, as well as prevention and treatment of infections.

Decline in neonatal mortality might have been hampered by insufficient feedback mechanisms within the hospital or between the hospital and peripheral health facilities where patients are referred from. Therefore, all deaths occurring in the hospital whether maternal or newborn should be reviewed and discussed between obstetricians and paediatricians; the cause identified, preventive measures worked out, and feedback given to all the staff involved within the institution as well as surrounding health facilities through existing outreach programs.

Regular review of neonatal deaths using simple classifications within a particular setting will help to understand the magnitude of the problem and review the strategies for better improvement. Reviews should be directed towards identifying areas where screening, prevention and therapeutic interventions need to be strengthened.

Efforts should be done to further develop registration systems in order to collect information that can be used to understand maternal and newborn health outcomes and serve as guidance for further preventive measures.

## Competing interests

The authors declare that they have no competing interests.

## Authors’ contributions

BTM: Study design, methodology, data analysis and manuscript writing. RTL, RO, MJM, GK, AKD: Study design, methodology, manuscript writing. RTL and AKD approved the manuscript for final submission. All authors approved the final manuscript.

## Pre-publication history

The pre-publication history for this paper can be accessed here:

http://www.biomedcentral.com/1471-2431/12/116/prepub
